# Supplementary material for: Price elasticity of demand for ready-to-drink sugar-sweetened beverages in Brazil
Source: PLoS One. 2023 Nov 1;18(11):e0293413. doi: 10.1371/journal.pone.0293413 (PMC10619800; doi:10.1371/journal.pone.0293413)
Supplement: S1 Table — (DOCX) [file pone.0293413.s001.docx]

**Supporting information**

**S1 Table. Descriptive statistic of price and quantity acquired in households**

| Group | Price | | Quantity | |
| --- | --- | --- | --- | --- |
|  | Mean | Std. Dev. | Mean | Std. Dev. |
| Ready to drink SSB | 3.15 | 0.002 | 0.92 | 0.018 |
| Diet Soda | 3.47 | 0.003 | 0.03 | 0.003 |
| Whole Juice | 10.18 | 0.014 | 0.12 | 0.008 |
| Prepared SSB | 24.69 | 0.029 | 0.15 | 0.005 |
| Dairy Beverages | 10.34 | 0.005 | 0.25 | 0.005 |
| Energy drink | 10.36 | 0.030 | 0.01 | 0.001 |
| Milk | 7.13 | 0.027 | 1.79 | 0.029 |
| Coffee and tea | 27.71 | 0.051 | 0.21 | 0.004 |
| Water | 0.96 | 0.002 | 1.74 | 0.061 |
| Ice Cream | 11.57 | 0.007 | 0.05 | 0.002 |
| Sweets | 22.20 | 0.042 | 1.05 | 0.002 |
| Snacks and Pizza | 22.99 | 0.013 | 0.07 | 0.004 |
| Bakery | 14.37 | 0.014 | 0.23 | 0.003 |
| Other foods | 12.89 | 0.140 | 12.45 | 0.095 |

Source: Own elaboration.
