# Supplementary material for: Price elasticity of demand for ready-to-drink sugar-sweetened beverages in Brazil
Source: PLoS One. 2023 Nov 1;18(11):e0293413. doi: 10.1371/journal.pone.0293413 (PMC10619800; doi:10.1371/journal.pone.0293413)
Supplement: S2 Table — (DOCX) [file pone.0293413.s002.docx]

**Supporting information**

| **S2 Table. Estimated probit model for the occurrence of consumption of each category of products in households** | | | | | | | | | | | | | | |
| --- | --- | --- | --- | --- | --- | --- | --- | --- | --- | --- | --- | --- | --- | --- |
| Explanatory variables | Ready-to-drink SSB | Diet Soda | Juice | Prepared SSB | Dairy Beverages | Energetics | Milk | Coffee and Tea | Water | Ice cream | Candies | Snacks and Pizza | Bakery | Other foods |
| Intercept | -1.924*** | -5.205*** | -2.682*** | -3.361*** | -4.757*** | -4.210*** | -3.359*** | -0.991*** | -3.429*** | -3.602*** | -3.505*** | -1.377*** | -1.909*** | -1.272*** |
| Tornqvist | -0.396*** | -0.061 | -0.140*** | 0.929*** | 1.176*** | -0.093 | 1.669*** | 0.484*** | -0.113*** | -0.568*** | 0.146*** | -0.190*** | 0.265*** | 2.743*** |
| Urban | 0.151*** | 0.168* | 0.073** | 0.085*** | 0.137*** | 0.118 | -0.006 | -0.041** | 0.390*** | 0.053 | 0.035 | 0.057*** | 0.046** | -0.025 |
| Region -North (reference) |  |  |  |  |  |  |  |  |  |  |  |  |  |  |
| North East | -0.087*** | -0.175 | -0.730*** | -0.118*** | 0.073*** | -0.230** | 0.010 | -0.103*** | 0.320*** | -0.071 | -0.068** | -0.220*** | 0.228*** | -0.017 |
| Midwest | 0.116*** | -0.036 | -0.976*** | 1.56*** | 0.060** | 0.112 | -0.032 | -0.226*** | -0.571*** | 0.163*** | 0.115*** | -0.089*** | -0.119*** | -0.444*** |
| Southeast | 0.078*** | 0.280** | -0.982*** | 0.226*** | 0.195*** | 0.065 | -0.023 | -0.257*** | -0.394*** | 0.119** | 0.181*** | 0.178*** | 0.152*** | -0.258** |
| South | 0.319*** | 0.213* | -0.930** | 0.291*** | 0.363*** | 0.271*** | 0.075*** | -0.015 | -0.285*** | 0.191*** | 0.501*** | 0.** | 0.176*** | -0.059 |
| Women | -0.073*** | 0.025 | 0.003 | 0.008 | 0.032** | 0.031 | 0.008 | 0.001 | 0.010 | -0.001 | 0.056*** | -0.043** | 0.002 | 0.042 |
| Years of study | 0.013*** | 0.014** | 0.024*** | 0.007*** | 0.018*** | 0.024*** | 0.003** | -0.003* | 0.021*** | 0.021*** | 0.016*** | 0.019*** | 0.012*** | -0.009* |
| Credit card | 0.024** | 0.054** | 0.066*** | 0.012 | 0.033*** | 0.070*** | -0.042*** | -0.005 | 0.047*** | 0.012 | 0.036*** | 0.019* | 0.024*** | 0.000 |
| Race -White (reference) |  |  |  |  |  |  |  |  |  |  |  |  |  |  |
| Black or brown | -0.074*** | -0.221*** | -0.004 | 0.018 | -0.041** | -0.101** | -0.044*** | -0.019 | -0.093*** | -0.024 | -0.071*** | -0.053*** | -0.028** | -0.026 |
| Asian or Indigenous | 0.057 | -0.089 | 0.077 | 0.073 | 0.027 | -0.089 | 0.033 | 0.126** | -0.139 | -0.077 | 0.091 | -0.109 | -0.036 | -0.225 |
| Residents from 0 to 5 years old | 0.054*** | -0.265*** | 0.044** | 0.101*** | 0.230*** | -0.064 | 0.126*** | -0.050*** | -0.010 | 0.034 | 0.042*** | 0.040** | 0.112*** | -0.204*** |
| Residents 6 to 12-year-old | 0.064*** | -0.111** | 0.047*** | 0.098*** | 0.122*** | 0.058 | 0.087*** | 0.027*** | -0.038** | 0.057*** | 0.042*** | 0.041*** | 0.117*** | 0.061* |
| Residents 13 to 18-year-old | 0.070*** | -0.064 | -0.012 | 0.122*** | 0.044*** | -0.066 | 0.052*** | 0.062*** | -0.052*** | 0.012 | 0.032*** | 0.026* | 0.071*** | 0.082** |
| Residents 19 to 59-year-old | 0.066*** | -0.061** | 0.003 | 0.050*** | 0.021** | 0.038 | 0.054*** | 0.093*** | -0.015 | 0.000 | 0.022*** | 0.027*** | 0.029*** | 0.122*** |
| Residents 60-year-old or more | -0.030** | -0.015 | -0.005 | 0.001 | -0.036** | -0.060 | 0.099*** | 0.124*** | 0.011 | -0.052** | -0.020 | -0.075*** | 0.011 | 0.144*** |
| Log of per capita income | 0.132*** | 0.282*** | 0.231*** | 0.064*** | 0.137*** | 0.198*** | 0.001 | -0.014 | 0.194*** | 0.200*** | 0.189*** | 0.204*** | 0.085*** | -0.041 |
| Number of rooms | -0.011*** | 0.017 | 0.004 | -0.013** | -0.004 | -0.020 | 0.001 | 0.002 | -0.007 | 0.003 | 0.009** | 0.015*** | 0.012*** | -0.002 |
| Piped water | 0.136*** | 0 | -0.154*** | 0.050 | 0.205*** | 0.193 | 0.107*** | -0.109*** | 0.018 | 0.258* | 0.009 | 0.051 | -0.089** | 0.006 |
| Sewage | 0.011 | 0.119* | 0.006 | -0.012 | -0.028 | -0.028 | -0.034** | -0.048*** | 0.067*** | 0.003 | 0.010 | 0.052** | -0.030** | -0.022 |
| Oven | 0.035* | 0.067 | -0.042 | 0.011 | 0.049** | 0.101* | 0.020 | -0.004 | -0.004 | 0.075** | 0.104*** | 0.082*** | 0.059*** | 0.000 |
| Stove | 0.053 | 0.120 | 0.114 | -0.168** | 0.078 | -0.188 | 0.206*** | -0.040 | 0.077 | -0.013 | 0.141 | -0.112 | 0.053 | 0.334** |
| Refrigerator | 0.153*** | -0.086 | -0.310*** | 0.076 | 0.117** | -0.128 | 0.090** | -0.074* | 0.072 | 0.227* | 0.084 | 0.147* | 0.039 | -0.025 |
| Freezer | 0.030 | -0.035 | 0.058** | -0.029 | 0.015 | -0.122* | 0.031* | 0.048*** | -0.007 | -0.025 | 0.014 | -0.111*** | -0.060*** | -0.0031 |
| Chi-square test | 2097.36*** | 521.43*** | 2137.39*** | 1971.17*** | 3960.86*** | 295.83*** | 4469.47*** | 1020.20*** | 2189.58*** | 608.62*** | 2815.53*** | 2040.12*** | 1193.31*** | 2132.45*** |
| Pseudo R^2^ | 0.0417 | 0.1547 | 0.1091 | 0.0541 | 0.0891 | 0.0894 | 0.0700 | 0.0179 | 0.0884 | 0.0571 | 0.0717 | 0.0779 | 0.0203 | 0.3544 |

Source: Own elaboration.
