# Supplementary material for: Price elasticity of demand for ready-to-drink sugar-sweetened beverages in Brazil
Source: PLoS One. 2023 Nov 1;18(11):e0293413. doi: 10.1371/journal.pone.0293413 (PMC10619800; doi:10.1371/journal.pone.0293413)
Supplement: S3 Table — (DOCX) [file pone.0293413.s003.docx]

**Supporting information**

**S3 Table. Goodness of fit measures for QUAIDS model.**

| Equation | RSME | R^2^ | MAE | F-test |
| --- | --- | --- | --- | --- |
| Ready to drink SSB | 0.064 | 0.192 | 0.032 | 400.837*** |
| Diet Soda | 0.005 | 0.028 | 0.001 | 48.593*** |
| Whole Juice | 0.035 | 0.198 | 0.011 | 416.456*** |
| Prepared SSB | 0.032 | 0.068 | 0.012 | 123.075*** |
| Dairy Beverages | 0.041 | 0.131 | 0.019 | 254.290*** |
| Energy drink | 0.006 | 0.008 | 0.001 | 13.604*** |
| Milk | 0.114 | 0.189 | 0.067 | 393.115*** |
| Coffee and tea | 0.065 | 0.164 | 0.036 | 330.914*** |
| Water | 0.051 | 0.130 | 0.016 | 252.059*** |
| Ice Cream | 0.023 | 0.120 | 0.006 | 230.026*** |
| Sweets | 0.043 | 0.073 | 0.016 | 132.838*** |
| Snacks and Pizza | 0.051 | 0.046 | 0.016 | 81.337*** |
| Bakery | 0.072 | 0.201 | 0.038 | 424.533*** |
| LogLikehood | 1189311 | | | |
| AIC | -2378050 | | | |
| BIC | -2375544 | | | |

Source: Own elaboration.
