# Supplementary material for: Price elasticity of demand for ready-to-drink sugar-sweetened beverages in Brazil
Source: PLoS One. 2023 Nov 1;18(11):e0293413. doi: 10.1371/journal.pone.0293413 (PMC10619800; doi:10.1371/journal.pone.0293413)
Supplement: Attachment — Submitted filename: Comments PONE-D-23-03690.docx [file pone.0293413.s005.docx]

The paper discusses an interesting issue of policy relevance; Demand Price Elasticity for Ready-To-Drink Sugar-Sweetened Beverages in Brazil. While the paper is well delivered, I provide some comments for improvement.

1. The term ‘Demand Price Elasticity’ should be checked in title.
2. What are the contribution of the research to the literature, policy planners and consumers should add to the abstract.
3. Introduction needs some data of how much percentage of SSB consumption increased between 2009 and 2014 in Latin American countries. Similarly how much percentage of risk of developing obesity and diseases increased in?
4. How taxation policy impacted on price of sugar-sweetened beverages need some explanation for general reader in introduction.
5. Page 7 in Introduction paragraph 2 Drop in productivity of what?
6. Research objectives/research questions are not well defined, expenditure and income spent on SSB by type of SSB and subgroups are omitted to be belonging to the objectives.
7. The rationale and applicability of applying the Quadratic Almost Ideal Demand System (QUAIDS) methods needs to be further explained.
8. Where is the estimated probit models? Please give the equation with empirical model, and variable explanation is better placing in a table instead of description. And please also provide the steps where consumption probabilities were inserted into the estimation of the demand system and the subsequent calculation of elasticities.
9. Page 13, 2^nd^ paragraph, what does it mean ‘24.05% of the families’? Is it surveyed families, please make it clear.
10. The data and analyses are well described.
11. In concluding section (last paragraph) please correlate the tax with price and add a strong policy implication based on findings.
12. Please provide the data of prices and annual demand (if possible, otherwise amount of consumption) for all SSB in 2017-2018 as supplementary.
13. Finally, the language needs further polishing.
