# Supplementary material for: Price elasticity of demand for ready-to-drink sugar-sweetened beverages in Brazil
Source: PLoS One. 2023 Nov 1;18(11):e0293413. doi: 10.1371/journal.pone.0293413 (PMC10619800; doi:10.1371/journal.pone.0293413)
Supplement: Attachment — Submitted filename: Response to Reviewers.docx [file pone.0293413.s007.docx]

Dear editor,

We would like to thank you for the time spending evaluating our paper. The modifications are highlighted in the article (file labeled 'Revised Manuscript with Track Changes').

**Editor comment**

Although the paper has been improved, one of the reviewers has asked for a major correction on your model. Also, the necessary model’s diagnostic tests and goodness of fit measures are still missing. Thus, I invite you for a second revision to address those concerns.

**Answer:** *Thanks for your comment.* *We have done the corrections in our model, as proposed by the reviewer, and present the results with the adjustments. Also, we present table S3 with goodness of fit measures, the table shows that the model presented a good quality of fit, with low values for RSME and MAE, and good values of R^2^, considering the characteristics of the database, with F test indicating global significance of all equations of the system, information criteria were also reported.*

**Reviewer 1 Comment**

Reviewer #1: The authors have done a great job addressing most of my previous comments. For this round, I only have concerns about the prices they use for estimation. In the revised draft, they use unit values as individual prices, which is worrisome. In estimating demand system, you want to avoid using unit values as possible as you can since they have quality effects embedded in them and thus are correlated with preferences making them endogenous. For example, for individuals who tend to buy premium RTD SSBs versus others who buy average brands, the former would pay much higher unit values than the latter for the same amount of SSBs consumed, reflecting individual's preferences. Please see Nelson (1991) and Nelson (1990) that discuss the issue rather well.

One approach you might want to consider in order to fix this issue is to look at those sub-products within the aggregate group of RTD SSBs, such as sodas, soft drinks, nectars etc. Calculate unit values for these sub-products and use these sub-group unit values to construct a Laspeyres or Paasche type index for the aggregate group RTD SSBs. The idea is to start with the most elementary prices in the data set and construct the indexes for the aggregates. It will be less of a problem if you start with the disaggregate unit values and construct index numbers based on them the aggregate group of RTD SSBs.

**Answer:** *Thanks for your comment. We really appreciate it. We have done the quality-adjustment in the unit values. To do this, we follow the suggested strategy to use indexes for the aggregated category and calculated them as the expenditure divided by total quantity consumed in the category, weighted by expenditure share. Also, we clarify how to we have treated the missing values for non-consuming households. Please, see the changes in lines 219-224. The new results with the quality-adjusted unit value are shown in Results section.*

Do not hesitate to contact us if any other information is needed at this stage of the process.

Best regards,

Authors
