# Supplementary material for: Price elasticity of demand for ready-to-drink sugar-sweetened beverages in Brazil
Source: PLoS One. 2023 Nov 1;18(11):e0293413. doi: 10.1371/journal.pone.0293413 (PMC10619800; doi:10.1371/journal.pone.0293413)
Supplement: Attachment — Submitted filename: Response to Reviewers.docx [file pone.0293413.s008.docx]

Dear editor,

We would like to thank you for the time spending evaluating our paper. The modifications are highlighted in the article (file labeled 'Revised Manuscript with Track Changes').

**Editor comment**

Please discuss the limitation of your study in the conclusion by focusing on the weaknesses of your study such as positive own price elasticity of some items, low R-squared value…etc. Also, provide suggestions and recommendation for future research.

**Answer:** *Thanks for your comment.* *Your recommendations and insights were extremely helpful in enhancing our work. All suggested changes have been incorporated into the revised article. We believe these alterations have significantly strengthened the work, making it more robust and relevant to Plos One's readership. Please, see the changes in lines 471-486.*

Please do not hesitate to reach out to us should there be any additional information or clarification needed.

Sincerely,

The authors
